# Case Report: Shifting the paradigm in refractory in-stent restenosis: systemic immunomodulation with hydroxychloroquine and sirolimus

**DOI:** 10.3389/fcvm.2026.1886312

**Published:** 2026-10-28

**Authors:** Jiangquan Liao, Yan Wang, Jie Zhang, Tianshu Xie, Peng Yang, Lin Zhao, Tong Gao, Qian Wang, Jiejia Liang, Qian Li, Weidong Hong, Ke Dong, Changhong Zhao, Di Wang, Xianlun Li, Mingjing Shao

**Affiliations:** 1 Department of Integrative Medicine Cardiology, China-Japan Friendship Hospital, Beijing, China; 2 Graduate School, Beijing University of Chinese Medicine, Beijing, China

**Keywords:** hydroxychloroquine, in-stent restenosis, refractory, sirolimus, systemic therapy

## Abstract

**Background:**

Recurrent in-stent restenosis (ISR) refractory to repeated percutaneous interventions remains a formidable clinical challenge. Despite the established efficacy of drug-coated balloons (DCBs) and drug-eluting stents, a subset of patients experience relentless restenosis, highlighting the need for novel systemic strategies targeting underlying pathophysiological drivers.

**Objectives:**

To describe a case of extreme, refractory recurrent ISR that failed multiple DCB interventions but was associated with a marked response to systemic therapy with Hydroxychloroquine (HCQ) and Sirolimus, and to discuss the mechanistic rationale and potential clinical implications.

**Methods:**

A 56-year-old female presented with rapidly recurring ISR in both the left anterior descending (LAD) and right coronary arteries (RCA). Over a 6-year period, she underwent 11 interventional procedures at our center and at outside institutions. Sequential trials of oral anti-inflammatory agents failed to halt the restenosis cycle. In May 2023, HCQ was initiated, and Sirolimus was added in October 2023. Serial angiographic and intravascular ultrasound (IVUS) follow-up were performed.

**Results:**

Prior to systemic therapy, restenosis occurred every 6 months in both vessels. After initiating HCQ and Sirolimus, the RCA remained free of restenosis for over one year. The LAD, while requiring additional focal DCB treatments, showed a marked reduction in restenosis burden, with disease becoming highly localized. IVUS revealed neointimal hyperplasia with superimposed lipid-rich and fibrous plaques, without evidence of stent fracture or malapposition.

**Conclusions:**

In a patient with recurrent ISR, targeted systemic immunomodulation with HCQ and Sirolimus was associated with sustained vessel patency and reduced restenosis burden. This case provides a hypothesis-generating observation that addressing systemic inflammatory and proliferative drivers may repersent a potential salvage strategy for refractory recurrent ISR, warranting further prospective investigation.

## Introduction

In-stent restenosis (ISR) remains a significant challenge in interventional cardiology, with recurrent ISR representing a particularly difficult clinical scenario. While drug-coated balloons (DCBs), predominantly paclitaxel-coated, and newer-generation drug-eluting stents (DES) form the cornerstone of ISR management, a subset of patients experience rapid, refractory restenosis that defies these standard therapies (1, 2). The pathophysiology often involves a vicious cycle of neointimal hyperplasia driven by smooth muscle cell proliferation, frequently amplified by a persistent local inflammatory response (3). In cases of recurrent ISR, simply repeating local interventions—even with different device platforms—may fail if the underlying systemic biological drivers, such as a hyper-proliferative diathesis or excessive inflammation, are not addressed (4, 5). This report presents an illustrative case of extreme, recurrent ISR refractory to multiple iterations of paclitaxel-coated balloon angioplasty, in which a strategic shift to combination systemic therapy of with Hydroxychloroquine (HCQ) and Sirolimus was associated with a marked change in disease course. This report details the clinical outcome and discusses the mechanistic rationale that may inform future management strategies for refractory ISR.

## Case presentation

### Initial presentation and history

A 56-year-old female was referred to our center in May 2020 with a one-year history of intermittent chest tightness worsening over three months. Her coronary history began in August 2019 with PCI for a 90% LAD stenosis, followed one month later by PCI for a 95% RCA stenosis, both treated with DES implantation at an outside hospital. Notably, a 50% ISR in the LAD was noted during the second procedure. She experienced recurrent angina six months later, and ISR in the LAD was treated with a DCB (details unavailable). Despite aspirin, ticagrelor, and high-intensity statin therapy (rosuvastatin/ezetimibe), her symptoms progressed. She was a postmenopausal female with a body mass index of 24.5 kg/m^2^. She had no history of hypertension, diabetes, or autoimmune disease. There was no family history of premature coronary artery disease (first-degree relatives with events before age 55 in males or 65 in females). She was a lifetime non-smoker, reported no alcohol consumption, worked as a retired office clerk, and denied any definite history of allergies.

### Diagnostic work-up and initial intervention at our center (may 2020)

Given the unusually rapid recurrence pattern, a systemic autoimmune or inflammatory etiology was considered in the differential diagnosis. The following conditions were systematically excluded: systemic lupus erythematosus (antinuclear antibody, anti-dsDNA negative), rheumatoid arthritis (rheumatoid factor, anti-CCP negative), systemic vasculitis (ANCA, anti-GBM negative). The only abnormal finding was an isolated elevation of erythrocyte sedimentation rate (ESR, 33 mm/h, reference range: 0–20 mm/h). Vascular ultrasound showed atherosclerotic changes but no evidence of systemic vasculitis. This diagnostic approach was performed prior to any intervention at our center and guided the decision to proceed with coronary angiography and intervention rather than primary immunosuppressive therapy.

Coronary angiography revealed a total occlusion of the LAD distal to the previous stent and a critical 99% stenosis within the mid RCA stent, which provided collaterals to the LAD. A staged PCI was performed. After successful recanalization and pre-dilation of the LAD, severe atherosclerotic disease was noted both within and beyond the existing stent. To avoid focal edge restenosis and ensure full lesion coverage, a new-generation DES (SYNERGY, Boston Scientific) was implanted after adequate preparation with a scoring balloon. The same strategy was applied to the RCA (Figure 1).

**Figure 1:**
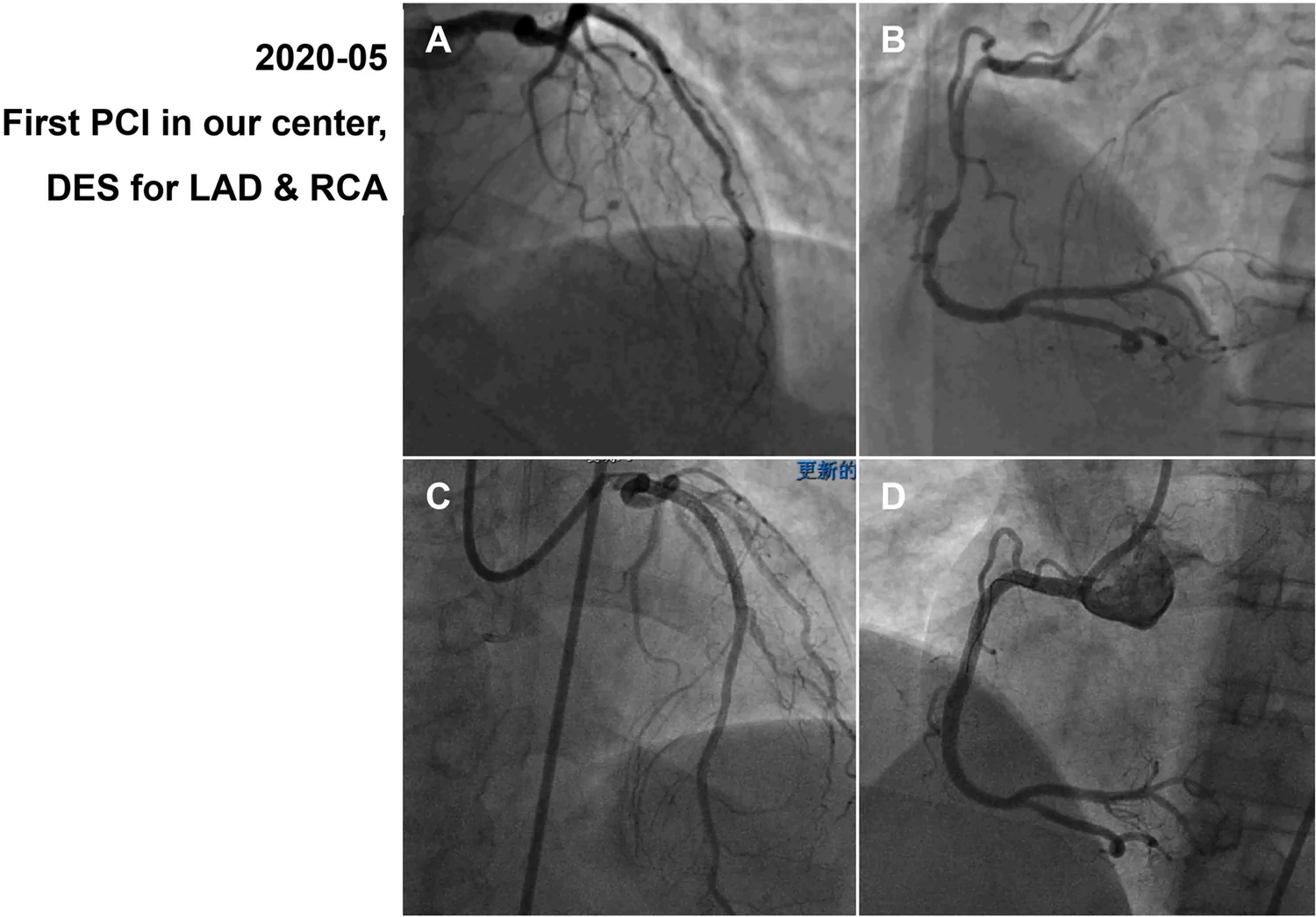
Coronary angiography in 2020−05. **(A)** LAD pre-intervention. **(B)** RCA pre-intervention. **(C)** LAD post-intervention. **(D)** RCA post-intervention.

### Cycle of recurrent ISR and escalating local therapy (nov 2020—apr 2024)

The patient subsequently entered a relentless cycle of recurrent, severe ISR in both vessels at approximately 6-month intervals (Table 1). Between November 2020 and April 2024, she underwent eight additional PCI procedures. A detailed review confirmed that all DCBs used during this period—including SeQuent® Please NEO (B. Braun), Bingo (Yinyi Biotech), SWIDE (Shenqi Medical), and Dissolve (DT MEDTECH)—were exclusively paclitaxel-coated (Table 2). Despite optimal lesion preparation, switching between different paclitaxel-DCB platforms, and achieving excellent acute results, the restenosis process continued unabated (Figures 1–3). LDL-C levels were consistently maintained at very low levels (<1.0 mmol/L for most of the period).

**Table 1:** Summary of PCI and systemic immunomodulatory therapy.

DATE	LAD	RCA	LDL-C (mmol/L)	Key Systemic Therapy	Fundametal Therapy
2019–08	DES*1 (detail unknown)	-	-	-	Aspirin, Clopidogrel, Atorvastatin, Ezetimibe
2019–09	-	DES*1 (detail unknown)	-	-	Aspirin, Ticagrelor, Atorvastatin, Ezetimibe
2019–11	DCB (detail unknown)	-	-	-	Aspirin, Ticagrelor, Atorvastatin, Ezetimibe
2020–05	SYNERGY（3.0 × 38mm）+SYNERGY（4.0 × 38mm）	SYNERGY（4.0 × 32mm）	1.91	-	Aspirin, Ticagrelor, Atorvastatin, Ezetimibe
2020–11	SeQuent® Please NEO（2.5 × 30mm）+SeQuent® Please NEO（3.0 × 20mm）	SeQuent® Please NEO（3.5 × 30mm）+SeQuent® Please NEO（3.5 × 26mm）	1.82	-	Aspirin, Ticagrelor, Atorvastatin, Ezetimibe
2021–05	Bingo（2.5 × 30mm）+Bingo（3.0 × 20mm）	Bingo（3.5 × 30mm）+Bingo（3.5 × 30mm）	1.09	-	Aspirin, Ticagrelor, Atorvastatin, Ezetimibe, Evolocumab
2021–11	Bingo（2.5 × 25mm）+Bingo（3.0 × 35mm）	Bingo（3.0 × 30mm）+Bingo（3.5 × 30mm）	2.98	Tripterygium Glycosides started	Aspirin, Ticagrelor, Atorvastatin, Ezetimibe, Evolocumab
2022–05	SWIDE（2.75 × 30mm）+SWIDE（3.0 × 30mm）	SWIDE（3.5 × 30mm）	0.87	Colchicine added	Aspirin, Ticagrelor, Atorvastatin, Ezetimibe, Evolocumab
2022–11	Bingo（3.0 × 35mm）+Bingo（3.0 × 35mm）	B.Braun（3.5 × 30mm）+B.Braun（3.5 × 26mm）+Bingo（3.5 × 25mm）	0.66	Tripterygium & Colchicine stopped	Aspirin, Ticagrelor, Atorvastatin, Ezetimibe, Evolocumab
2023–05	SWIDE（3.5 × 30mm）+SWIDE（3.5 × 25mm）	SWIDE（3.0 × 30mm）+Bingo（3.5 × 40mm）	0.35	HCQ started	Aspirin, Ticagrelor, Atorvastatin, Ezetimibe, Evolocumab
2023–10	SWIDE（3.0 × 25mm）+Dissolve（3.5 × 30mm）	SWIDE（3.0 × 30mm）+SWIDE（3.5 × 30mm）+SWIDE（4.0 × 30mm）	1.22	Sirolimus added & HCQ continued	Aspirin, Ticagrelor, Atorvastatin, Ezetimibe, Evolocumab
2024–04	SWIDE（3.5 × 30mm）、SWIDE（3.5 × 25mm）	Dissolve（3.5 × 30mm）+Dissolve（3.5 × 25mm）	0.57	HCQ & Sirolimus continued	Aspirin, Ticagrelor, Atorvastatin, Ezetimibe, Evolocumab
2024–12	Dissolve（3.5 × 35mm）+Dissolve（3.5 × 25mm）	50%–70%	0.66	HCQ & Sirolimus continued	Aspirin, Ticagrelor, Atorvastatin, Ezetimibe, Evolocumab
2025–11	Dissolve（3.5 × 30mm）	50%–70%	1.01	HCQ & Sirolimus continued	Aspirin, Ticagrelor, Atorvastatin, Ezetimibe, Evolocumab

**Table 2:** Details of DES and DCB.

	Brand Name	Eluting/coating drug	Manufacturer
DES	SYNERGY	Everolimus	BostonScientific
DCB	SeQuent® Please NEO	Paclitaxel	B. Braun
	Bingo	Paclitaxel	Yinyi Biotech
	SWIDE	Paclitaxel	Shenqi Medical
	Dissolve	Paclitaxel	DT MEDTECH

**Figure 2:**
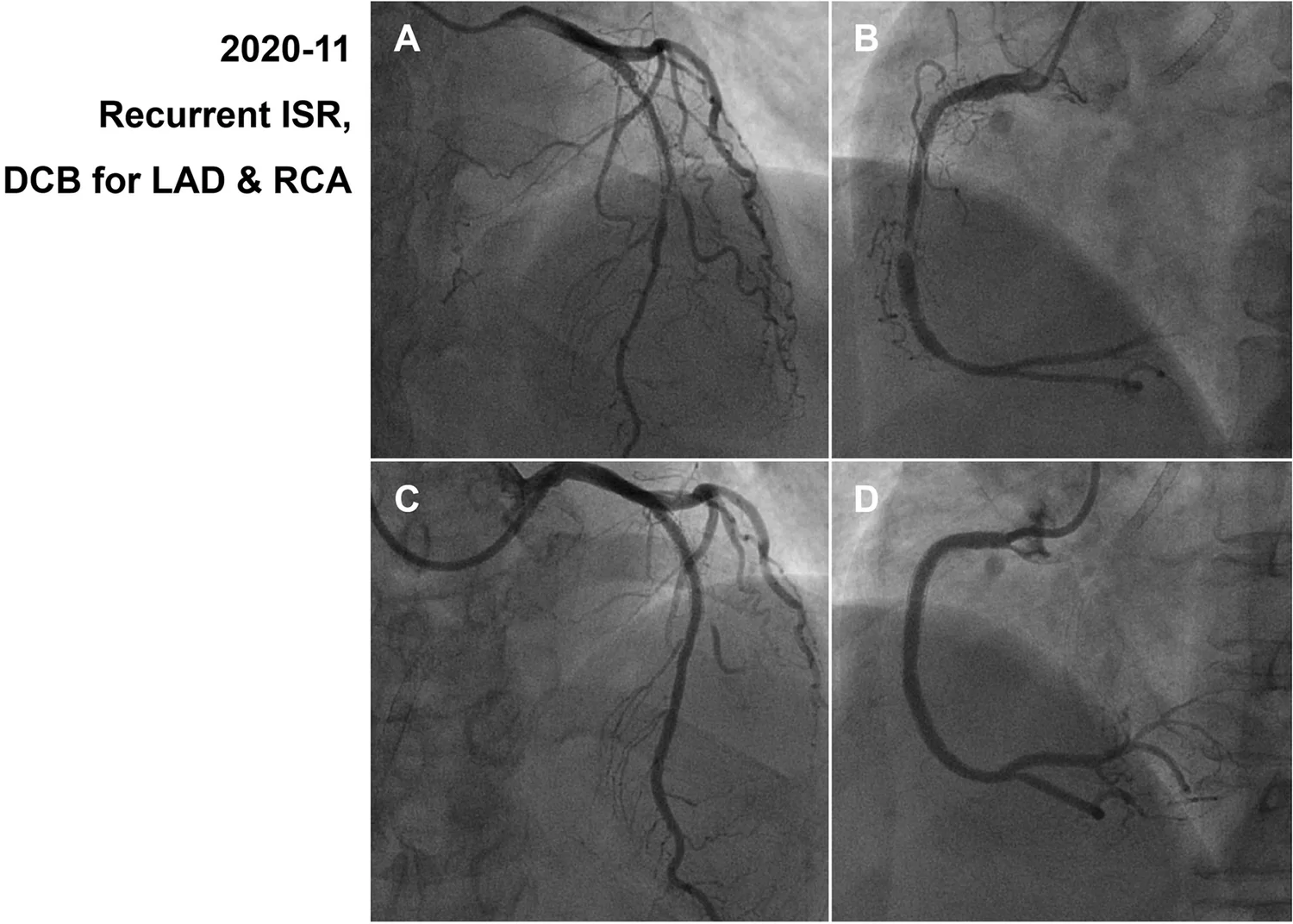
Coronary angiography in 2020−11. **(A)** LAD pre-intervention. **(B)** RCA pre-intervention. **(C)** LAD post-intervention. **(D)** RCA post-intervention.

**Figure 3:**
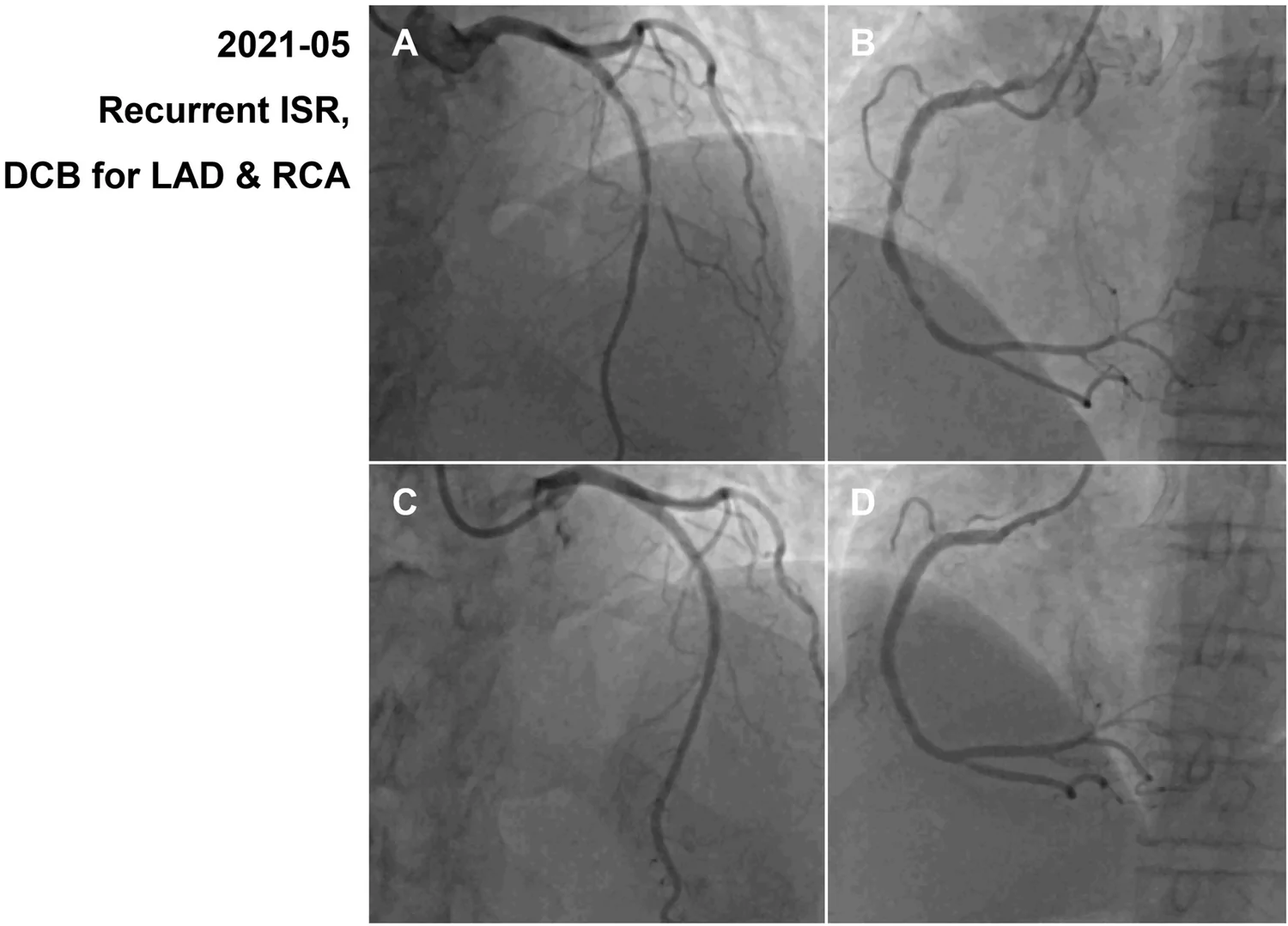
Coronary angiography in 2021−05. **(A)** LAD pre-intervention. **(B)** RCA pre-intervention. **(C)** LAD post-intervention. **(D)** RCA post-intervention.

### Transition to and response from systemic immunomodulatory therapy

Given the failure of repeated local paclitaxel-based therapy, a systemic approach was initiated:

1. Nov 2021: Tripterygium glycosides was added post-PCI without halting the cycle (Figure 4).
2. May 2022: Colchicine was combined with tripterygium glycosides, which also proved ineffective (Figures 5, 6).
3. May 2023: Therapy was switched to HCQ (200 mg bid). At the next intervention in October 2023, a modest reduction in LAD restenosis burden was noted. Sirolimus (1 mg qd) was subsequently added (Figures 7, 8).
4. Post-Combination Therapy (Apr 2024—Nov 2025): A marked change was observed. While both vessels required intervention in April 2024, the RCA ISR was notably focal and short, a significant deviation from previous diffuse patterns (Figure 9). By December 2024, angiography showed only 50%–70% stenosis in the RCA, which no longer required intervention, and focal ISR in the LAD (Figure 10). This stable RCA patency and focal LAD disease were maintained at the November 2025 follow-up (Figure 11). IVUS showed ISR with neointimal hyperplasia within the stents, with lipid-rich and fibrous plaques superimposed on this neointimal hyperplasia (Supplementary Video 1–5, Figures 12–15).

**Figure 4:**
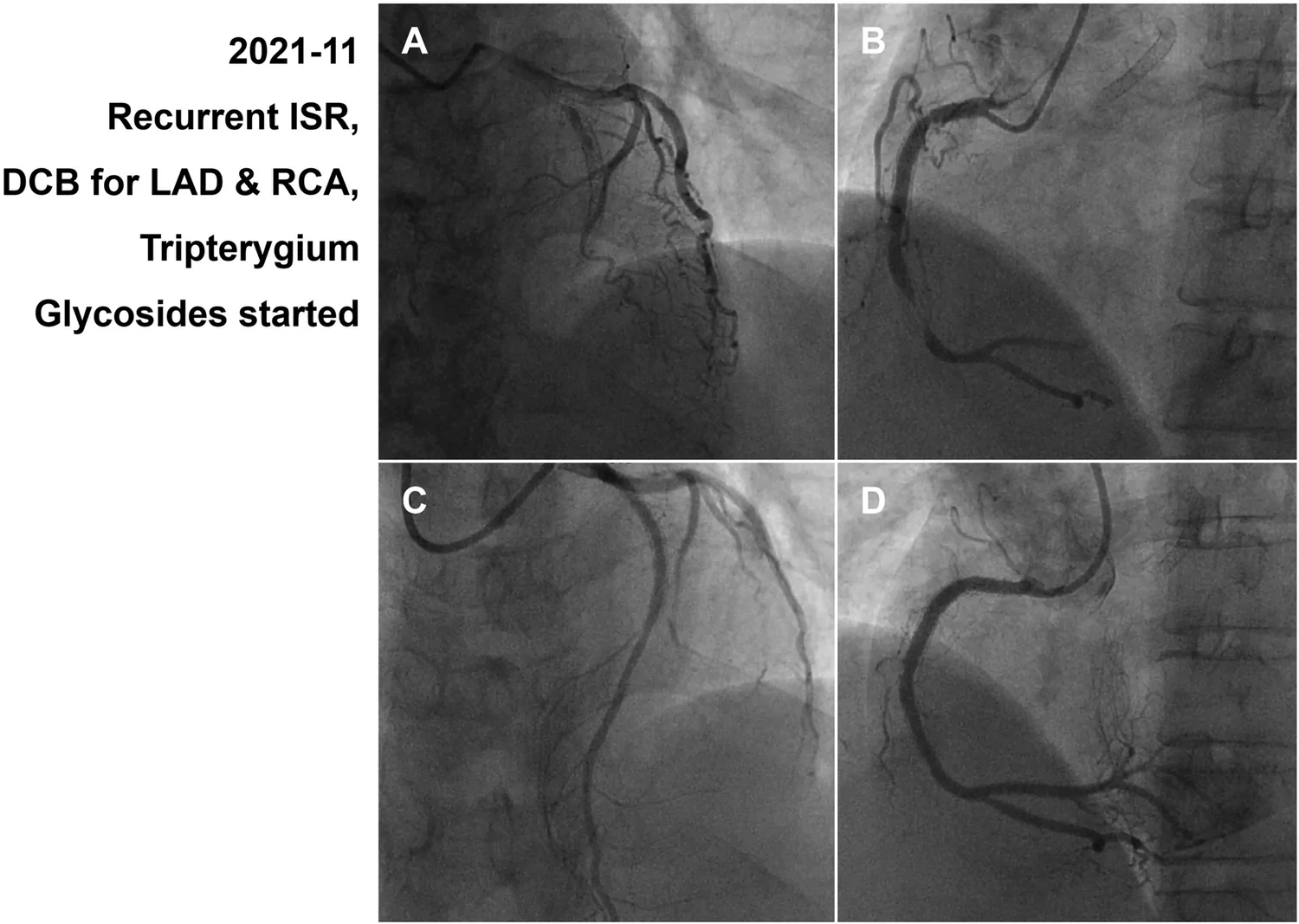
Coronary angiography in 2021−11. **(A)** LAD pre-intervention. **(B)** RCA pre-intervention. **(C)** LAD post-intervention. **(D)** RCA post-intervention.

**Figure 5:**
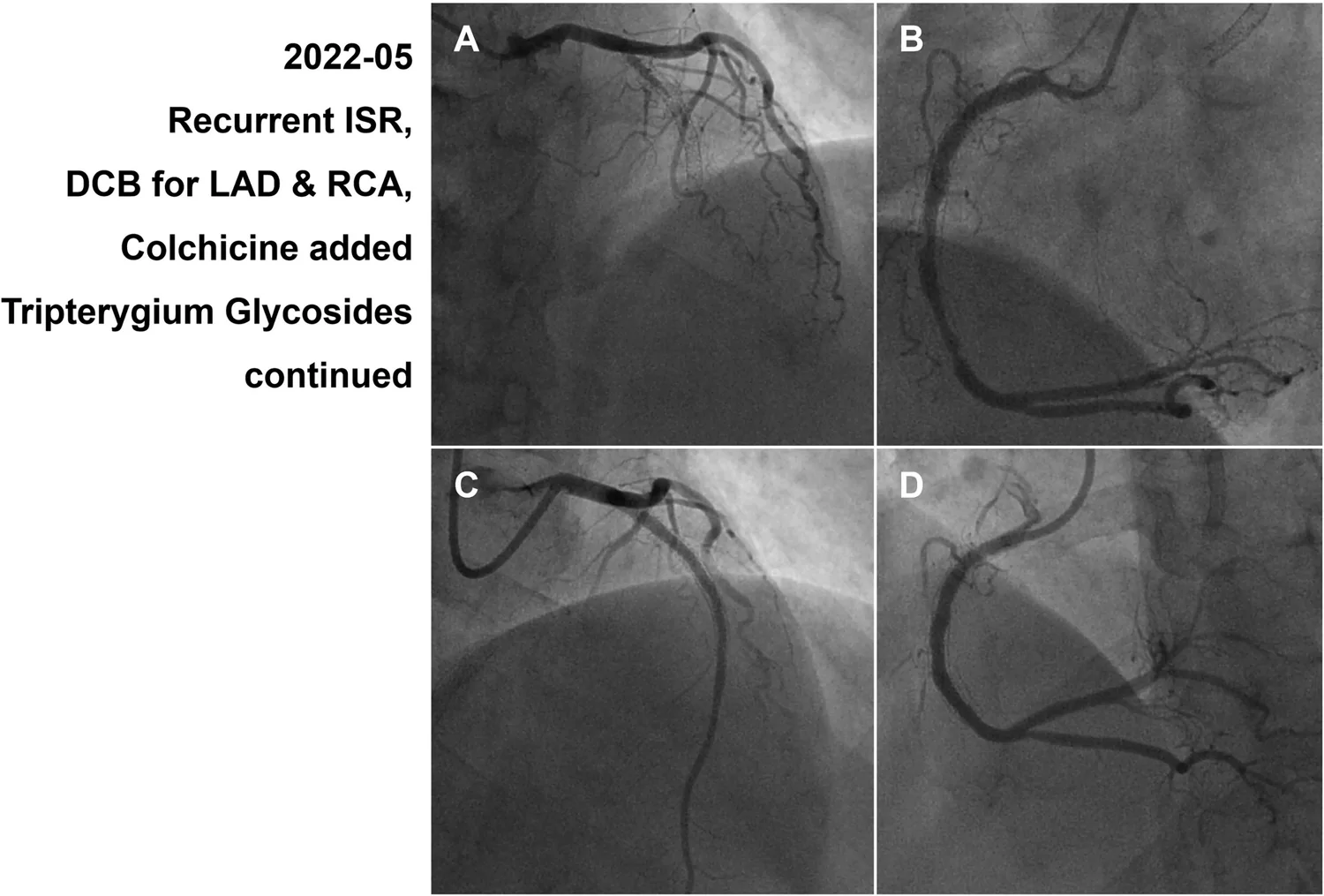
Coronary angiography in 2022−05. **(A)** LAD pre-intervention. **(B)** RCA pre-intervention. **(C)** LAD post-intervention. **(D)** RCA post-intervention.

**Figure 6:**
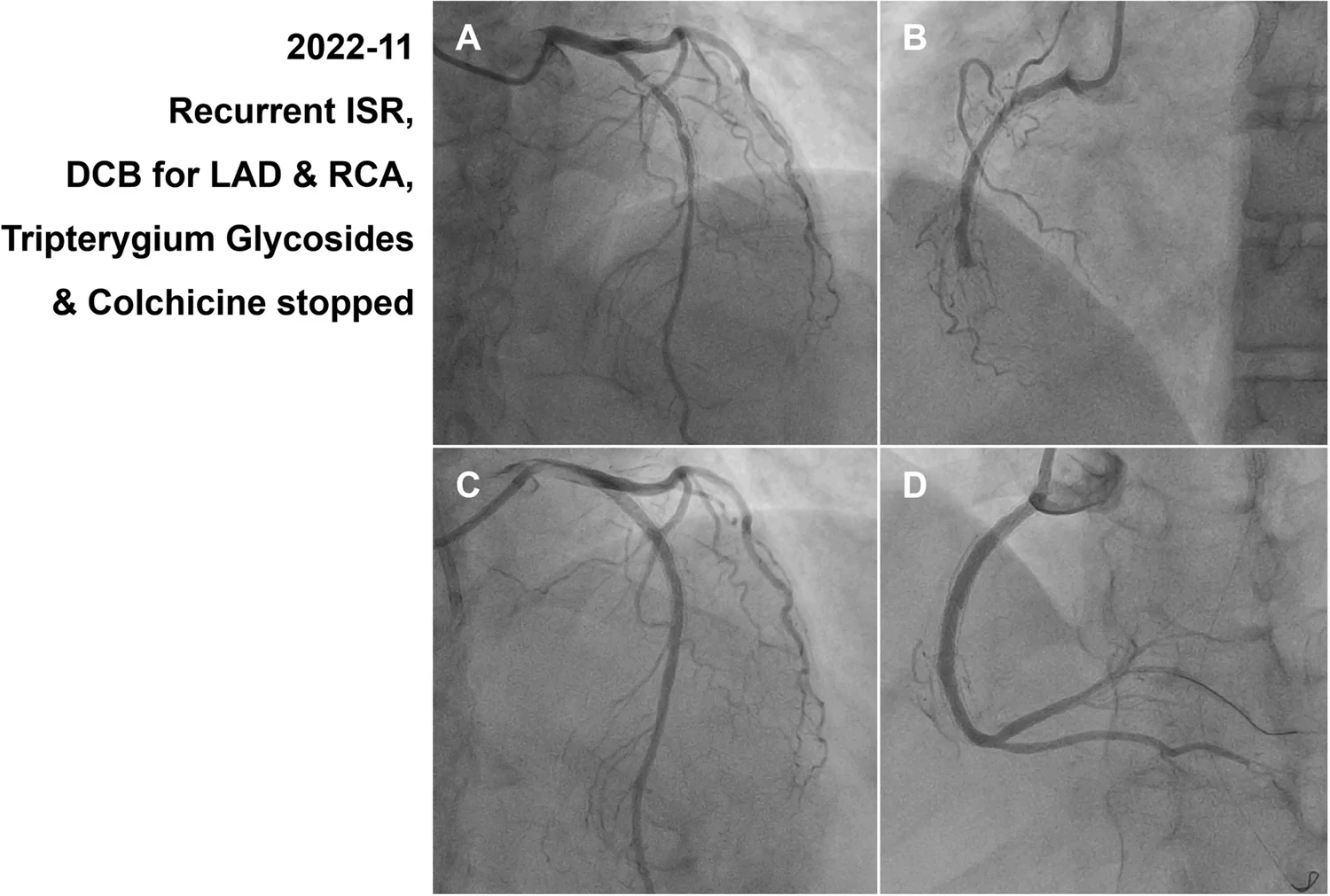
Coronary angiography in 2022−11. **(A)** LAD pre-intervention. **(B)** RCA pre-intervention. **(C)** LAD post-intervention. **(D)** RCA post-intervention.

**Figure 7:**
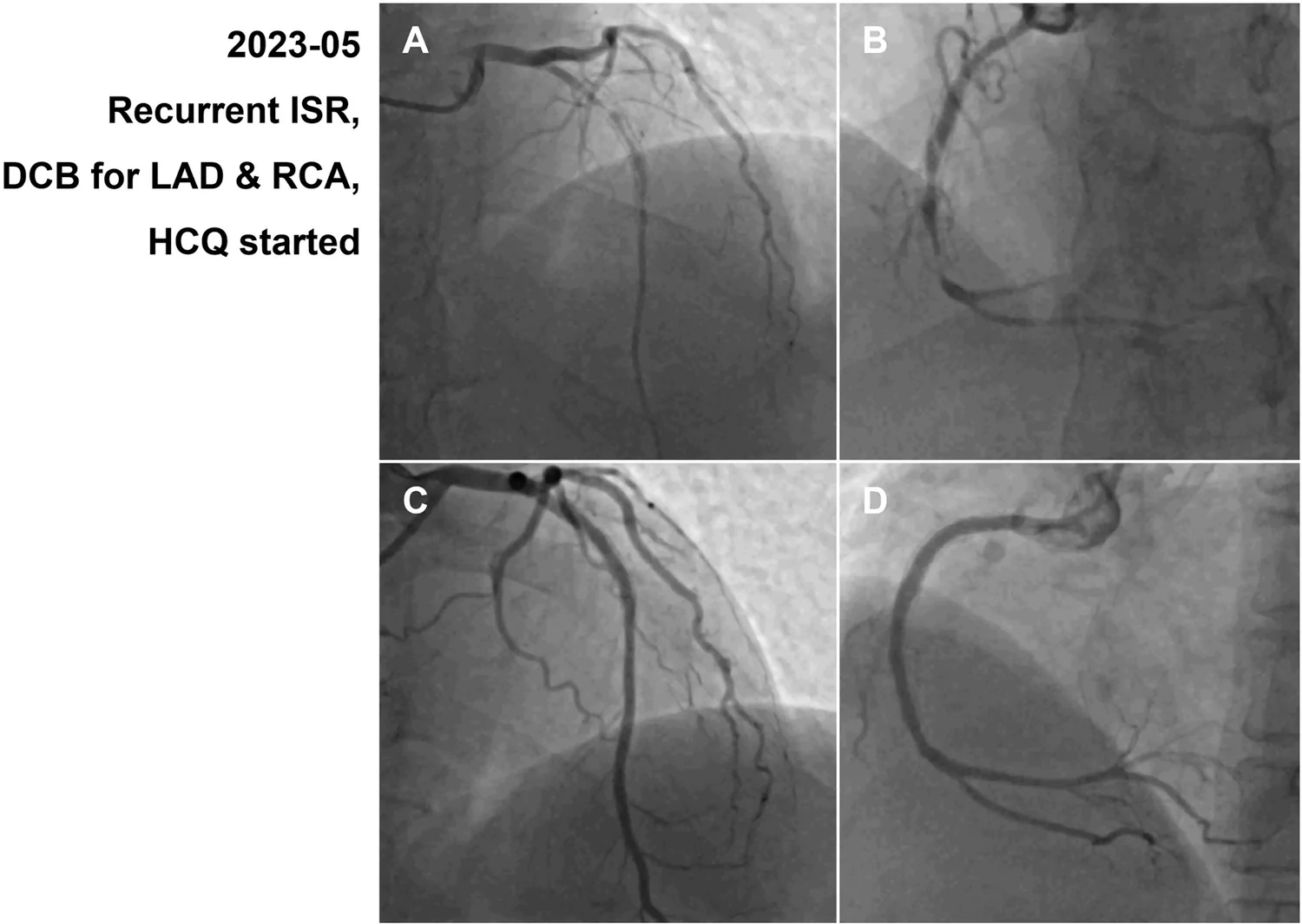
Coronary angiography in 2023−05. **(A)** LAD pre-intervention. **(B)** RCA pre-intervention. **(C)** LAD post-intervention. **(D)** RCA post-intervention.

**Figure 8:**
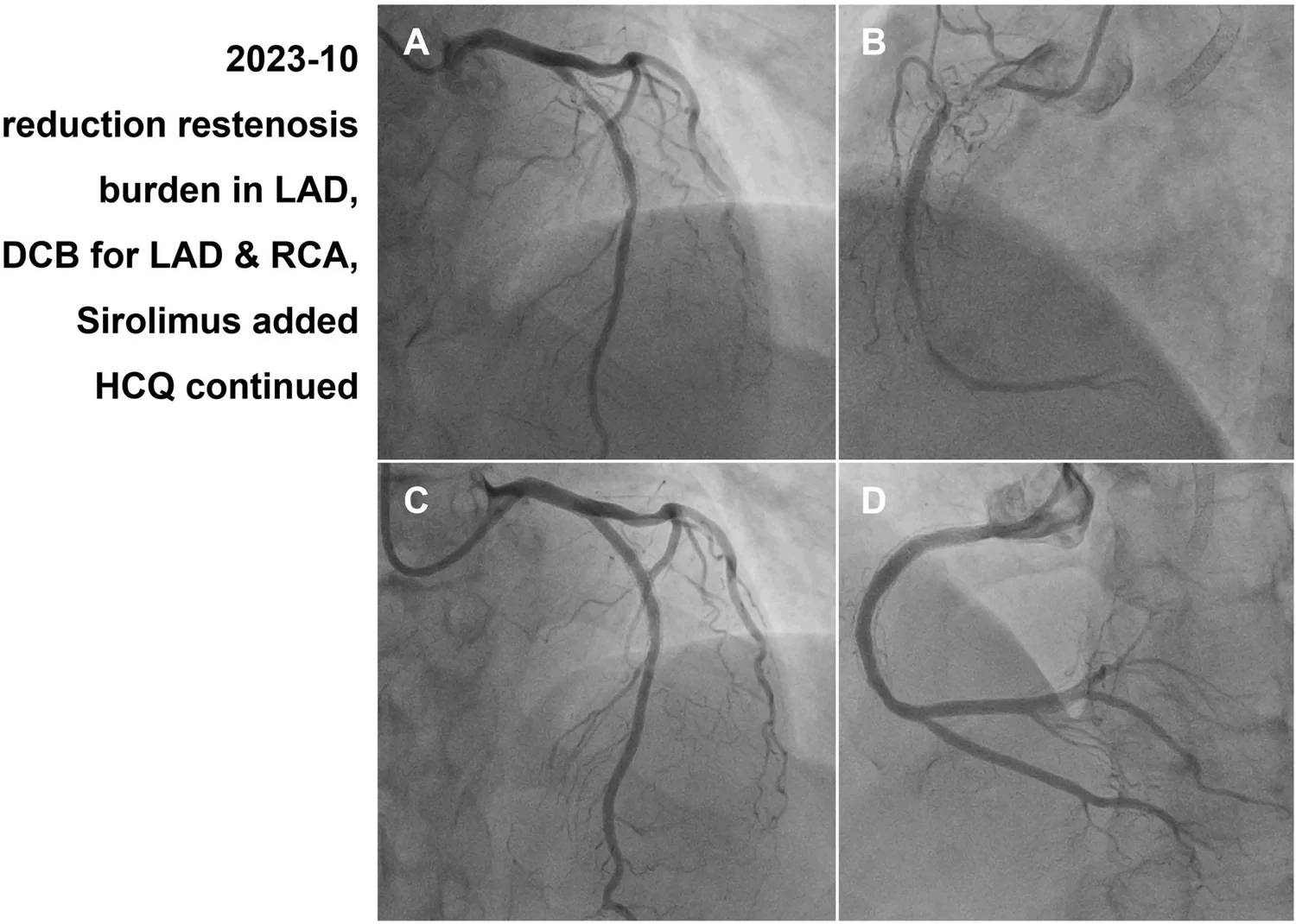
Coronary angiography in 2023−10. **(A)** LAD pre-intervention. **(B)** RCA pre-intervention. **(C)** LAD post-intervention. **(D)** RCA post-intervention.

**Figure 9:**
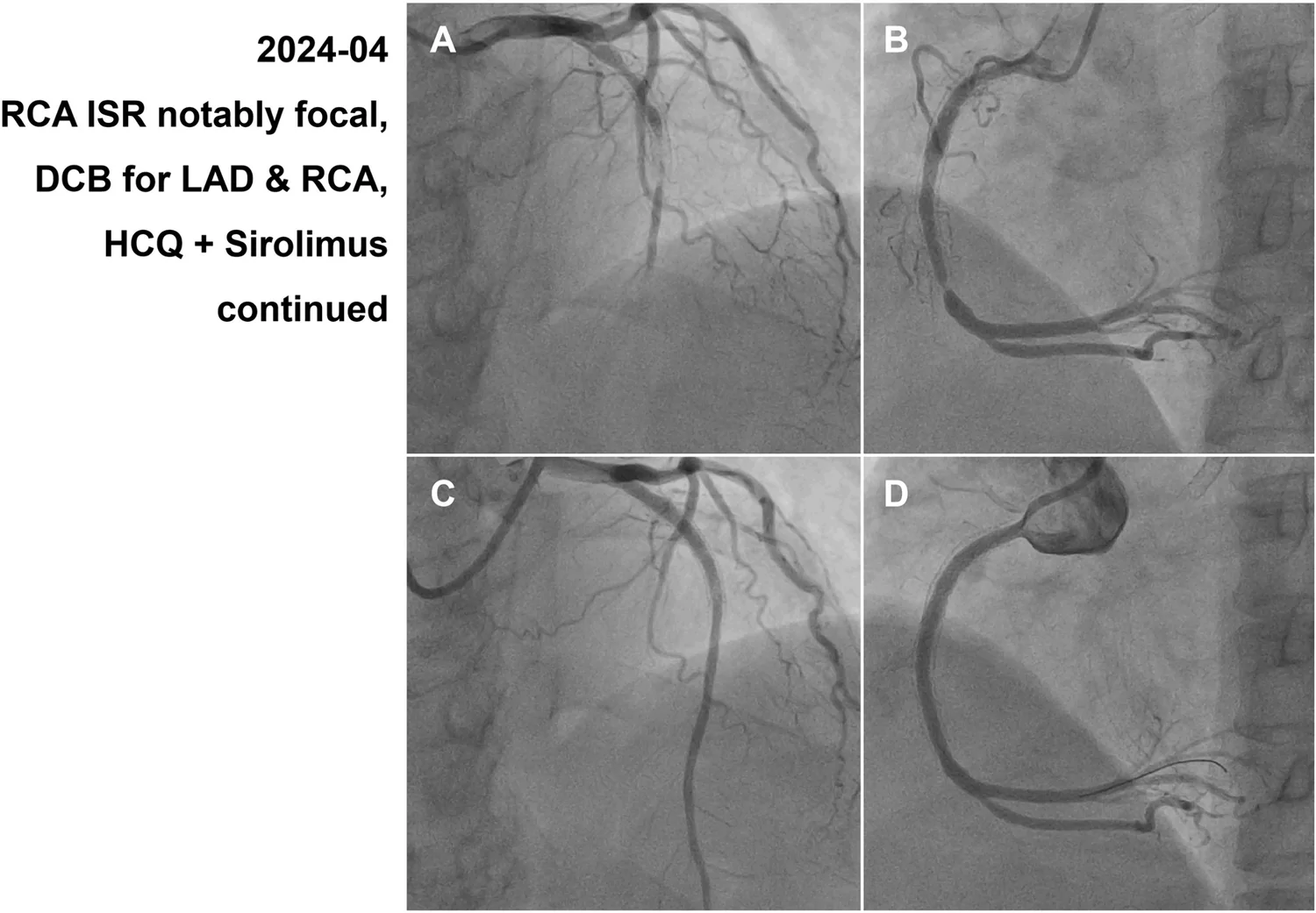
Coronary angiography in 2024−04. **(A)** LAD pre-intervention. **(B)** RCA pre-intervention. **(C)** LAD post-intervention. **(D)** RCA post-intervention.

**Figure 10:**
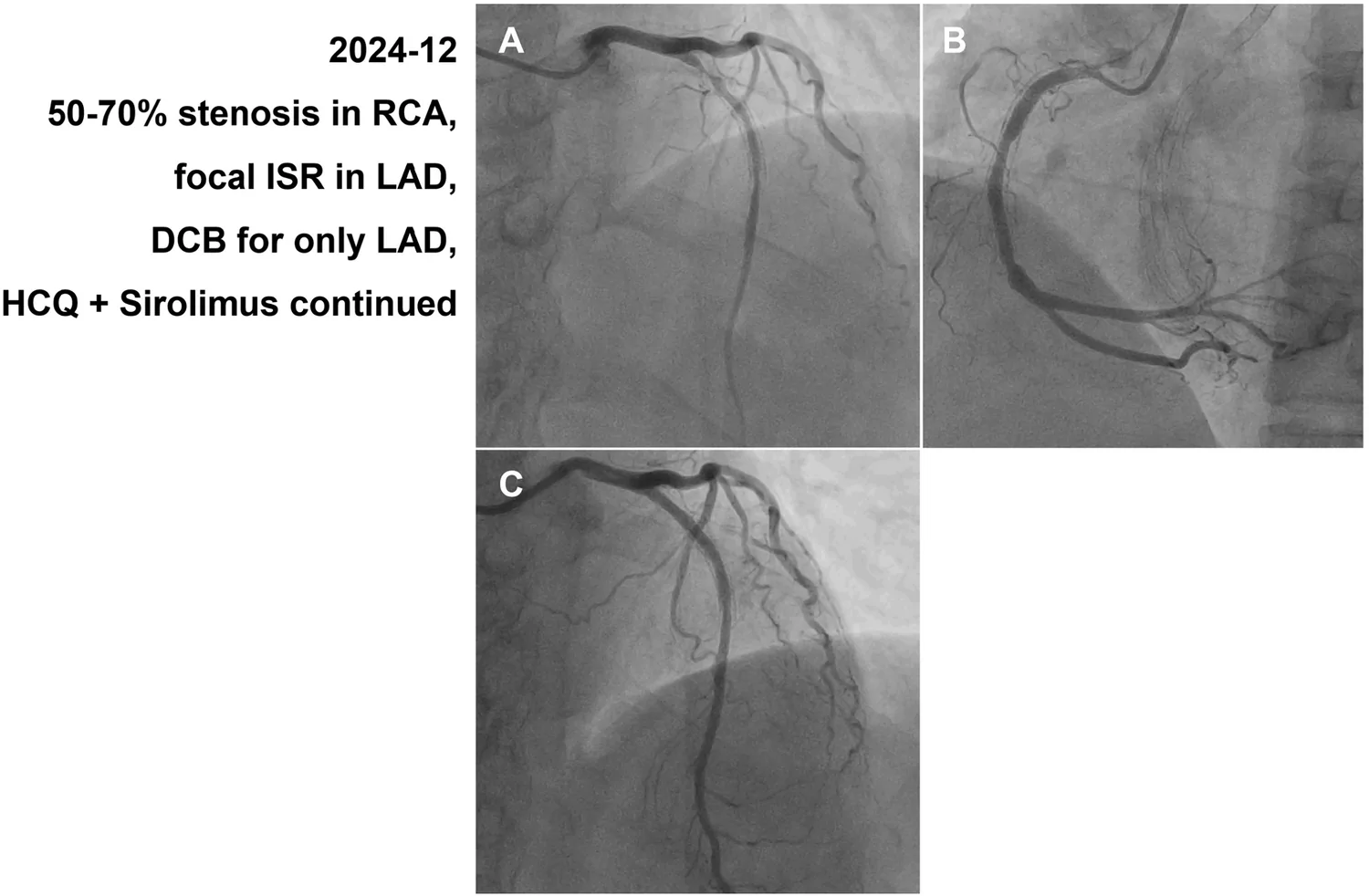
Coronary angiography in 2024-12. **(A)** LAD pre-intervention. **(B)** RCA pre-intervention. **(C)** LAD post-intervention.

**Figure 11:**
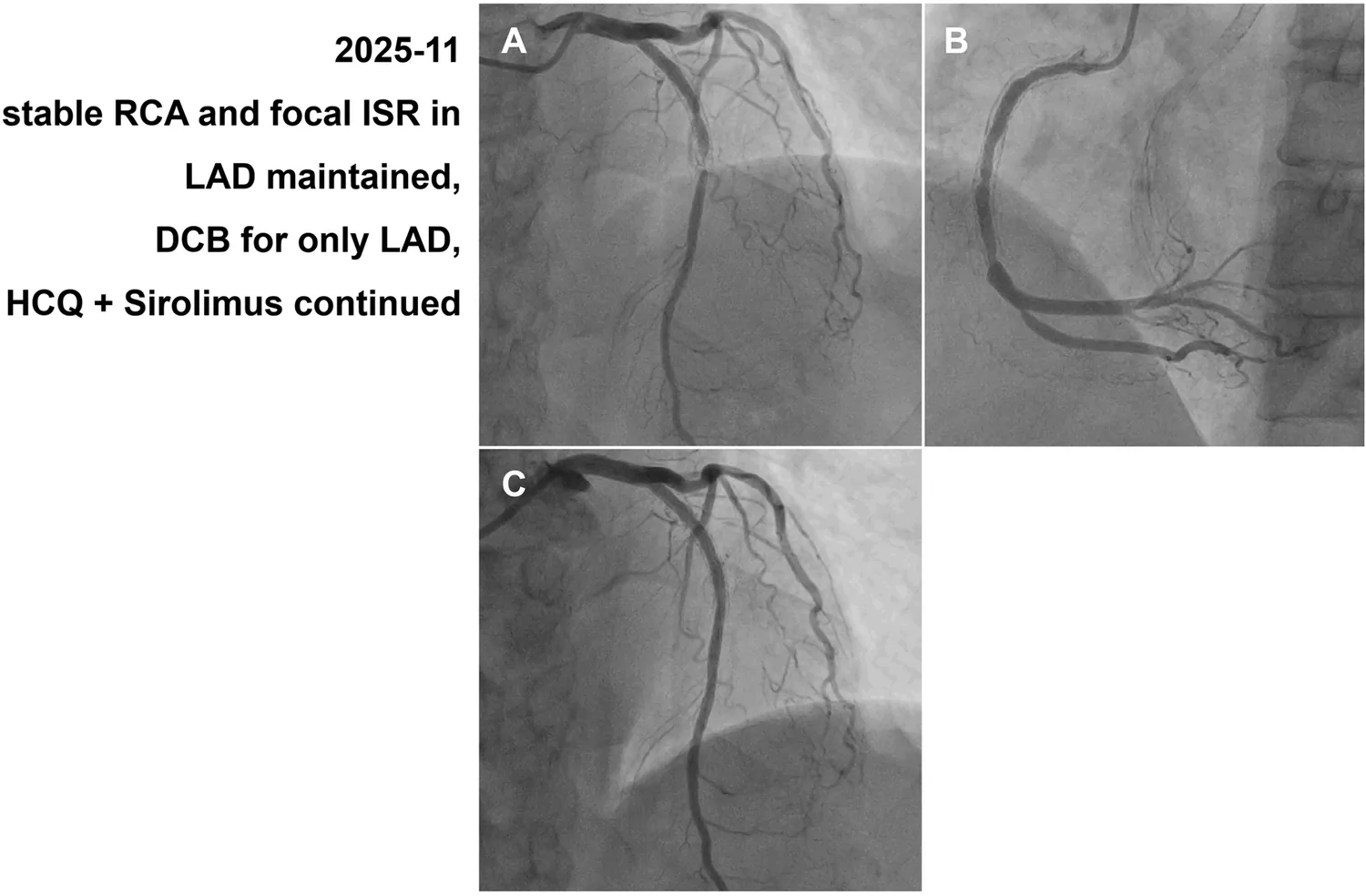
Coronary angiography in 2025−11. **(A)** LAD pre-intervention. **(B)** RCA pre-intervention. **(C)** LAD post-intervention.

**Figure 12:**
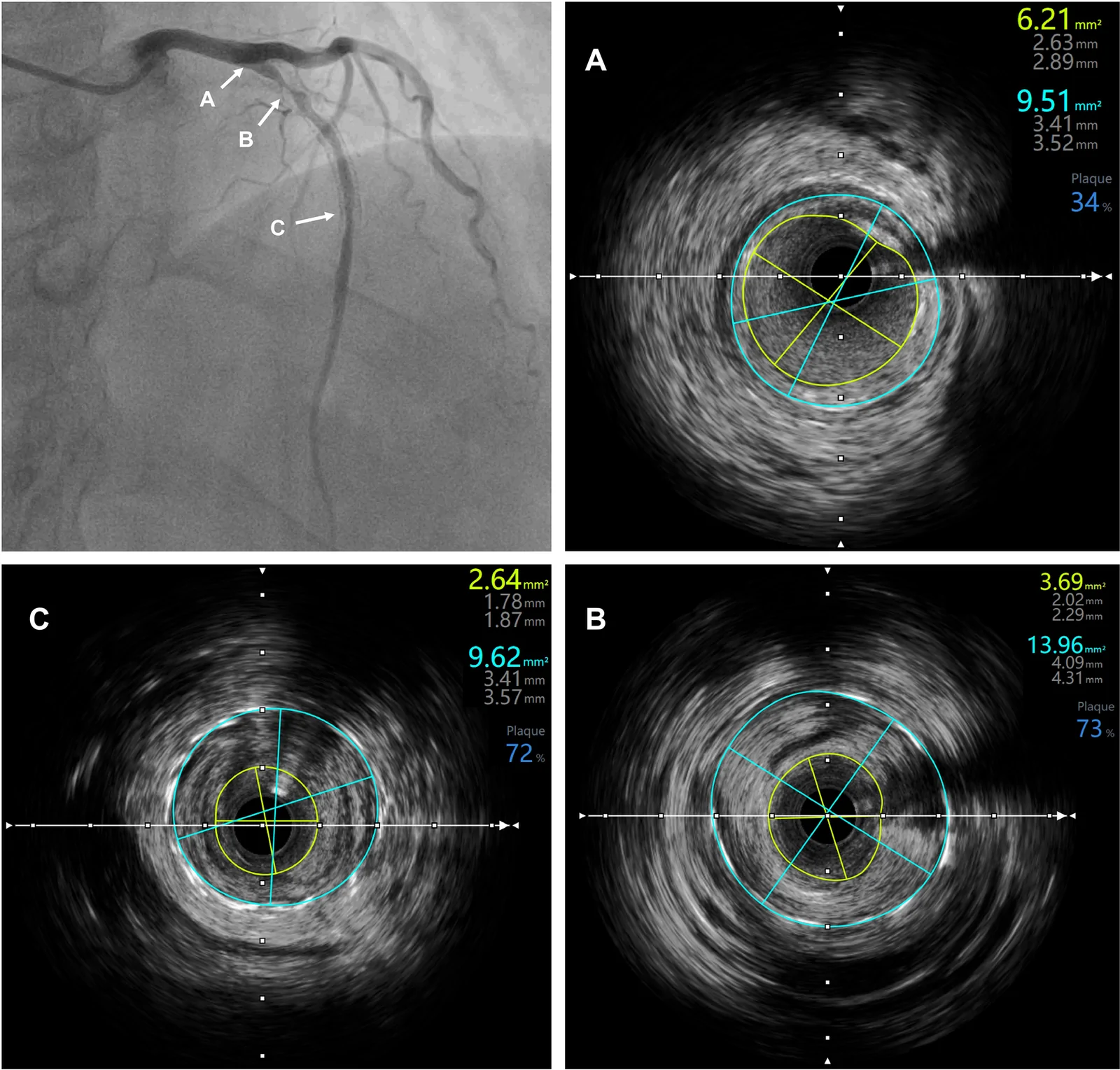
IVUS of LAD (post-dilation) in 2024–12–25. Different label letters **(A–C)** represent IVUS images of corresponding vessel locations.

**Figure 13:**
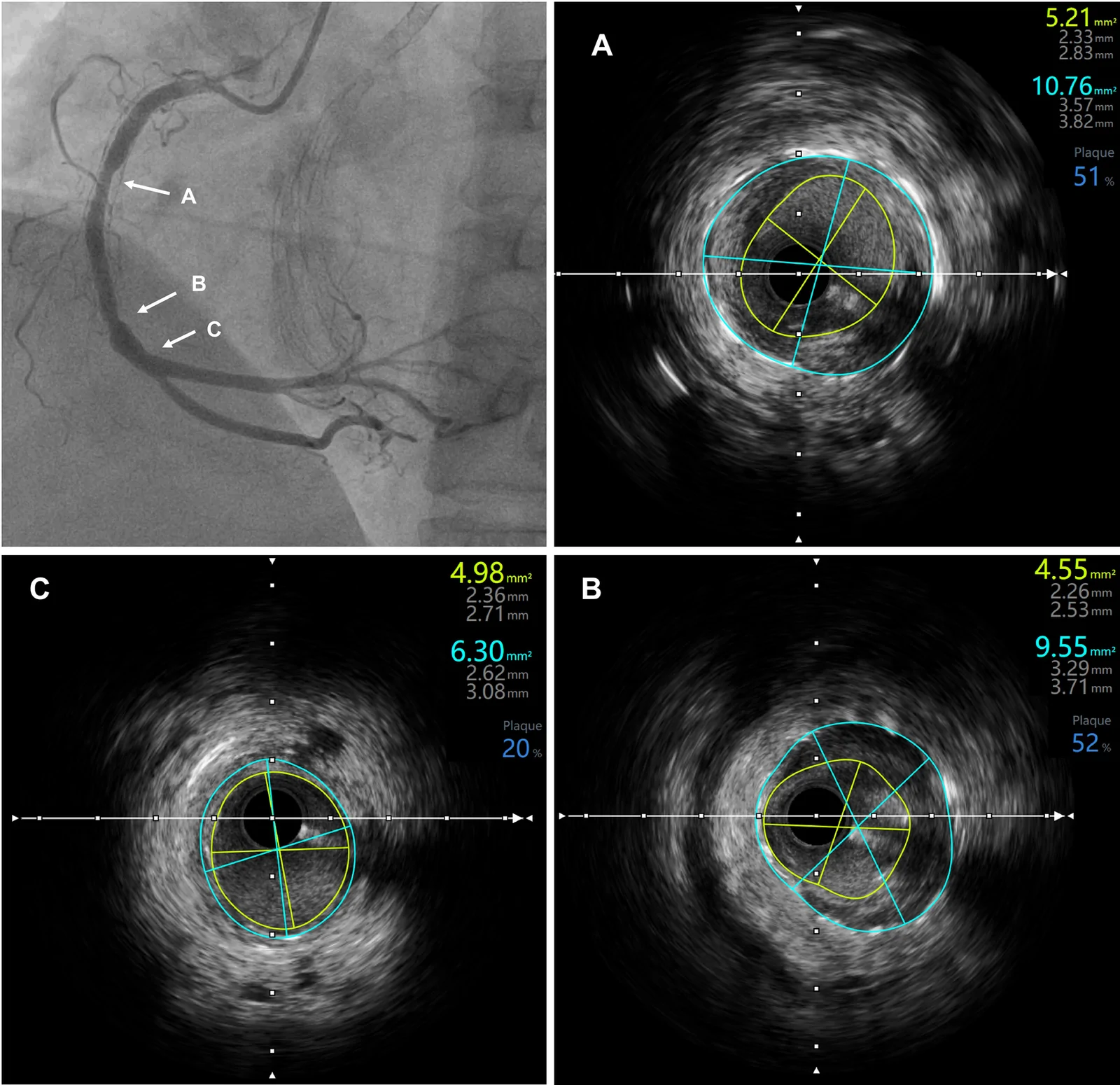
IVUS of RCA in 2024–12-25. Different label letters **(A–C)** represent IVUS images of corresponding vessel locations.

**Figure 14:**
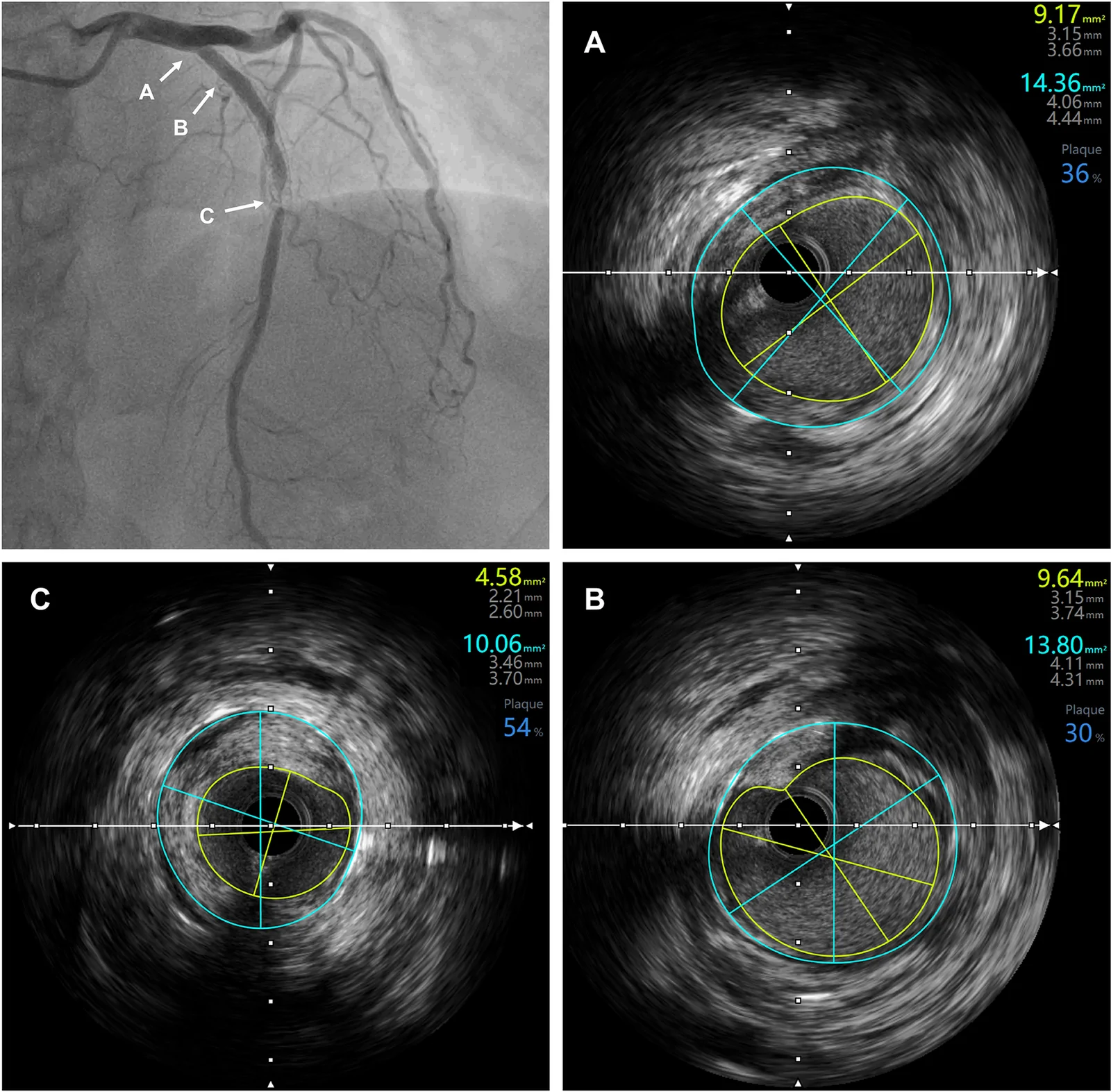
IVUS of LAD (post-dilation) in 2025-11-28. Different label letters **(A–C)** represent IVUS images of corresponding vessel locations.

**Figure 15:**
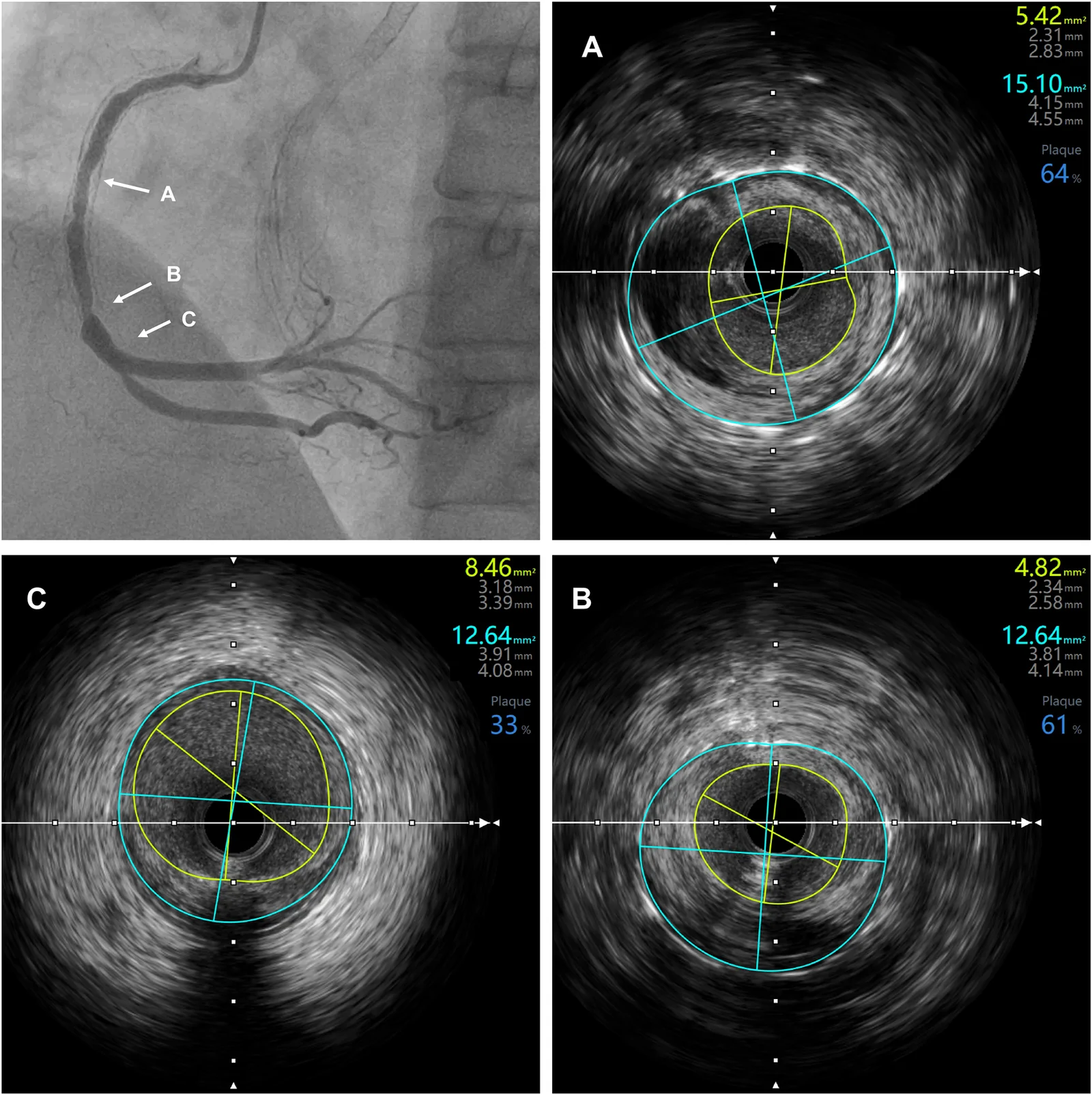
IVUS of RCA in 2025-11-28. Different label letters **(A–C)** represent IVUS images of corresponding vessel locations.

The patient remained on dual therapy with HCQ and Sirolimus without interruption through the most recent follow-up (July 2026). Treatment adherence was assessed by patient self-report at each follow-up visit, with adherence estimated at >95%.

The patient reported that prior to the initiation of HCQ and Sirolimus, her quality of life was severely limited by recurrent angina requiring repeated hospitalizations and procedures. Following the combination therapy, she experienced sustained relief from exertional chest pain and was able to resume normal daily activities without interruption for over one year.

Regular safety monitoring was performed every 3–6 months, including complete blood counts, liver and renal function tests, and fasting lipid and glucose profiles. No myelosuppression (leukopenia, thrombocytopenia, or anemia), hepatotoxicity, or significant metabolic disturbances were observed. Basic ophthalmic examination was performed annually at a local clinic thereafter, with no evidence of hydroxychloroquine-related retinopathy at the most recent assessment (November 2025).

## Discussion

This case presents a paradigm shift in the management of extremely refractory, recurrent in-stent restenosis (ISR). It illustrates the transition from exhaustive, repeated local therapies to a successful, mechanism-driven systemic strategy, highlighting a potential therapeutic pathway for a patient cohort with limited options.

### Summarizing the clinical challenge and outcome

The presented case is emblematic of the most challenging form of ISR. Despite guideline-adherent, aggressive local interventions—including repeat stenting and multiple applications of paclitaxel-coated balloons (PCBs) from various manufacturers—the patient experienced a relentless cycle of recurrent, severe restenosis at approximately 6-month intervals. This pattern persisted despite optimal procedural results and intensive lipid-lowering therapy, effectively defining a state of *refractoriness* to standard-of-care local antiproliferative therapy. The pivotal turning point was the initiation of combination systemic therapy with HCQ and Sirolimus. This strategy was associated with a marked alteration of the disease course, most notably evidenced by the sustained patency of the right coronary artery for over one year without intervention, and a marked reduction in the burden and severity of restenosis in both vessels.

### Pathophysiological insights into failure of local paclitaxel therapy

The patient's history underscores a critical limitation of the prevailing “local therapy-only” paradigm for complex ISR. The failure of multiple PCBs, despite their proven efficacy in many ISR cases (6, 7), suggests a biological milieu that overwhelms or evades the mechanism of paclitaxel (8). Paclitaxel exerts its effect by stabilizing microtubules, arresting smooth muscle cells (SMCs) and fibroblasts in the G2/M phase of the cell cycle and inhibiting proliferation (9). However, its effects are transient and localized.

Several factors may have contributed to this failure. First, the pathological substrate in recurrent ISR often evolves into a dense, fibrocellular hyperplasia that may limit drug penetration and uniformity (9). Second, a potent and persistent underlying inflammatory drive may continuously stimulate neointimal growth, outpacing the temporary suppression offered by a single PCB application (10). Finally, while head-to-head comparisons show overall similar efficacy between sirolimus- and paclitaxel-coated balloons for ISR (11), subtle mechanistic differences may be consequential in refractory cases. The presented case aligns with observations that the response to different antiproliferative agents can be heterogeneous and patient-specific (3, 11).

### Rationale for combined systemic immunomodulation and antiproliferation

The success of HCQ and Sirolimus combination therapy lies in its dual-pathway, systemic approach, fundamentally different from temporary local drug delivery.

#### Targeting the inflammatory driver with hydroxychloroquine

The initial failure of colchicine, another anti-inflammatory agent, and the subsequent success upon switching to HCQ is instructive. HCQ possesses broad immunomodulatory effects, including inhibition of Toll-like receptor signaling and cytokine production (e.g., IL-1β, IL-6), and modulation of antigen presentation. It is considered an antirheumatic drug with established cardiovascular benefit (12–14). We hypothesize that in this patient, HCQ effectively suppressed a specific, pervasive inflammatory signal that was fueling the hyper-proliferative response, a signal not fully addressed by colchicine or local therapy alone. This action may have attenuated the inflammatory milieu of the vascular microenvironment, rendering it less permissive to unrestrained neointimal growth.

#### Systemic vs. Local Antiproliferation with Sirolimus

The addition of Sirolimus represented the second critical pillar. Unlike a PCB's brief exposure, Sirolimus provides sustained systemic inhibition of the mTOR pathway, a central regulator of cell cycle progression, protein synthesis, and proliferation (15, 16). This continuous suppression from within the bloodstream may overcome limitations of local drug delivery by providing sunstained inhibitory pressure on circulating progenitor cells and SMCs throughout the coronary tree (17). The contrast between the mechanisms of the failed local therapy and the successful systemic therapy is summarized below:

The synergy between these agents is plausible: HCQ may dampen the inflammatory stimuli that activate proliferative pathways, while Sirolimus directly blocks the final common pathway of cellular proliferation, creating a comprehensive biological blockade against neointimal formation (Table 3).

**Table 3:** Comparison of local and systemic strategy.

Feature	Failed Local Strategy (Paclitaxel PCB)	Successful Systemic Strategy (HCQ + Sirolimus)
Primary Target	Microtubule assembly (cytoskeleton)	mTOR pathway (cell signaling & cycle) & Immune activation
Scope of Action	Focal, at site of balloon contact	Systemic, whole vascular bed and circulating cells
Duration of Action	Transient (weeks to months)	Continuous with daily dosing
Therapeutic Goal	Temporary arrest of local cell division	Sustained suppression of proliferation & inflammatory drivers

### Clinical implications and redefining the therapeutic paradigm

This case compels us to reconsider the management algorithm for recurrent ISR. After failure of two or more standard interventions (e.g., DCB or DES), the diagnosis of “refractory recurrent ISR” should be considered, prompting a search for systemic contributors. This observation raises the hypothesis that a trial of systemic immunomodulation may be warranted in such scenarios, especially when signs of systemic inflammation are present.

It represents a move from a purely *device-centered* approach to a *patient-centered*, *mechanism-based* strategy. Instead of repeatedly treating the symptom (the neointima) with increasingly complex local tools, we targeted the inferred underlying systemic dysfunction (persistent inflammation and a hyper-proliferative diathesis). This aligns with the broader movement toward personalized medicine in interventional cardiology.

### Study limitations and future directions

We acknowledge that the proposed inflammatory mechanism, while biologically plausible, remains a hypothesis derived from this single case. The elevated ESR alone is nonspecific and does not establish a systemic inflammatory driver as the cause of the patient's restenosis. Similarly, the proposed effects of HCQ on Toll-like receptor signaling and cytokine production, and of Sirolimus on circulating progenitor cells, are mechanistic inferences based on known pharmacology rather than direct experimental evidence from this case. These hypotheses should be tested in future studies with appropriate biomarker profiling or tissue-level analyses.

The conclusions are inherently limited by the nature of a single case report. The observed benefits, while marked, must be confirmed in larger, prospective studies. Furthermore, the optimal dosing and long-term safety of this specific combination in coronary artery disease patients require careful evaluation, monitoring for known risks such as retinopathy, myelosuppression, and metabolic effects, as was performed in this case.

Future research should focus on prospectively identifying patients with refractory ISR who are most likely to benefit from systemic therapy. This could involve detailed profiling of inflammatory biomarkers, genetic predispositions, or specific histological characteristics of retrieved atherectomy specimens. Randomized controlled trials comparing standard care versus standard care plus systemic immunomodulation in this high-risk population are the necessary next step to validate this approach.

## Conclusion

In conclusion, this case demonstrates that for a distinct, challenging phenotype of refractory recurrent ISR resistant to multiple PCB interventions, a strategic shift from exclusive local therapy to mechanism-based systemic immunomodulation can markedly alter the disease course. The combination of HCQ and Sirolimus, targeting both pervasive inflammation and dysregulated proliferation, was temporally associated with a marked attenuation of the rapid restenosis cycle, most notably in the RCA, and significantly prolonged the intervention-free interval. While derived from a single case and requiring validation in prospective studies, our experience provides a hypothesis-generating rationale that warrants further investigation in the management algorithm for highly selected patients with otherwise untreatable recurrent ISR. It raises the possibility that when focal therapies repeatedly fail, addressing the underlying systemic biological drivers may represent a potential strategy requiring prospective validation.

## Data Availability

The original contributions presented in the study are included in the article/Supplementary Material, further inquiries can be directed to the corresponding author.
